# Decoding the metabolomic responses of *Caragana tibetica* to livestock grazing in fragile ecosystems

**DOI:** 10.3389/fpls.2024.1339424

**Published:** 2024-02-26

**Authors:** Minghui He, Yanlong Han, Yong Gao, Min Han, Liqing Duan

**Affiliations:** ^1^ College of Forestry, Inner Mongolia Agricultural University, Hohhot, China; ^2^ College of Desert Science and Engineering, Inner Mongolia Agricultural University, Hohhot, China; ^3^ Inner Mongolia Hangjin Desert Ecological Position Research Station, Ordos, Inner Mongolia, China

**Keywords:** *Caragana tibetica*, grazing intensity, non-volatile metabolites, regeneration, stress response

## Abstract

The population of *Caragana tibetica*, situated on the edge of the typical grassland-to-desert transition in the Mu Us Sandy Land, plays a vital ecological role in maintaining stability within the regional fragile ecosystem. Despite the consistent growth of *C. tibetica* following animal grazing, the biological mechanisms underlying its compensatory growth in response to livestock consumption remain unclear. Analyzing 48 metabolomic profiles from *C. tibetica*, our study reveals that the grazing process induces significant changes in the metabolic pathways of *C. tibetica* branches. Differential metabolites show correlations with soluble protein content, catalase, peroxidase, superoxide dismutase, malondialdehyde, and proline levels. Moreover, machine learning models built on these differential metabolites accurately predict the intensity of *C. tibetica* grazing (with an accuracy of 83.3%). The content of various metabolites, indicative of plant stress responses, including Enterolactone, Narceine, and Folcepri, exhibits significant variations in response to varying grazing intensities (*P<*0.05). Our investigation reveals that elevated grazing intensity intensifies the stress response in *C. tibetica*, triggering heightened antioxidative defenses and stress-induced biochemical activities. Distinctive metabolites play a pivotal role in responding to stress, facilitating the plant’s adaptation to environmental challenges and fostering regeneration.

## 1 Introduction

Recent studies have indicated that the presence of shrubs in grasslands alters the light, temperature, moisture, and nutrient environments, leading to changes in species composition, community structure, and distinct ecological processes within shrub-encroached grasslands as compared to typical grasslands (Eldridge et al., 2011). *Caragana tibetica* exemplifies the adaptive resilience inherent in shrub-encroached grasslands within the Inner Mongolia Autonomous Region. *C. tibetica* showcases a distinctive umbrella-shaped structure characterized by sturdy, deep roots and well-defined taproots, fostering lateral root growth at specific depths (Zhang et al., 2009). Its well-developed root system, woody stems, and thorny structure contribute to its exceptional drought tolerance, enabling robust growth in these harsh environments, making it a vital forage for sheep in the Ordos region. In previous research conducted by our team on the conservation of rare and endangered plants, it was observed that long-term enclosure resulted in decline and even death of *C. tibetica*. However, *C. tibetica* that had been grazed by livestock exhibited greater health and stability compared to both the enclosed and ungrazed areas. This study hypothesizes that moderate grazing by livestock might stimulate the sprouting growth of branches, thereby enhancing population diversity and stability. Despite the consistent growth of C. tibetica following animal grazing, the biological mechanisms underlying its compensatory growth in response to livestock consumption remain unclear.

Plants exhibit a spectrum of herbivore resistance mechanisms, typically classified as tolerance and avoidance. A prominent aspect of tolerance is the ability for swift regeneration. When subjected to grazing, plants may strategically reallocate their limited nutritional resources to accelerate the repair and regeneration of damaged components (Timmis and Ramos, 2021). Research conducted on *Gentianella amarella* revealed that stem clipping interrupted the suppression regulated by apical dominance. Clipped plants exhibited a higher fruit yield in comparison to undamaged control plants. Interestingly, despite a 75% clipping, the rate of regrowth did not significantly diminish compared to the regrowth rate following 50% clipping (Huhta et al., 2003). Another aspect pertains to avoidance; certain plants activate defensive strategies when subjected to grazing, including the synthesis of anti-herbivory compounds (Hanley, 1998) or the formation of physical defense structures (Oesterheld and McNaughton, 1991). These defense mechanisms could influence plant growth, potentially facilitating accelerated recovery and regeneration under more severe damage conditions. Plant branches commonly contain tissues crucial for cell division and growth; disturbance to these growth points during grazing may diminish the plant’s regenerative capacity. Therefore, the extent of compensatory growth in plants following grazing is influenced by the nature of stress and the duration of recovery (Oesterheld and McNaughton, 1991). The grazing not only directly affects the survival and regeneration of plants but also profoundly influences the overall health of ecosystems and the livelihoods of communities reliant on these ecosystems. The detailed and comprehensive research on the physiological adaptation of *C. tibetica* in desert environments is still lacking (Ma et al., 2004).

Untargeted metabolomics enables the identification and quantification of thousands of metabolites, serving as indicators reflecting a plant’s stress responses and adaptive mechanisms. Xiang et al. discovered that 70% acetone extract of *C. tibetica* stems exhibited potent superoxide anion scavenging activity (Xiang et al., 2005). Metabolomics techniques have been extensively employed in plant studies to investigate temperature effects (Cook et al., 2004), water and salinity stress (Brosché et al., 2005), sulfur responses (Nikiforova et al., 2005), phosphorus dynamics (Hernández et al., 2007), oxidation processes (Baxter et al., 2007), heavy metal impacts (Le Lay et al., 2006), and various combinations of stressors (Rizhsky et al., 2004). Previous studies have predominantly focused on investigating the impact of varying grazing intensities across different regions on species richness, biodiversity, and plant regeneration (Liu et al., 2016). This study employs metabolomics techniques to analyze simulated grazed branches of *C. tibetica*, aiming to scientifically identify the role of livestock saliva in stimulating compensatory growth from pruned branches of *C. tibetica*. It seeks to elucidate the synergistic effects between livestock grazing and the growth patterns of *C. tibetica*, addressing the balance between the conservation of the *C. tibetica* population and grazing practices. The ultimate goal is to provide theoretical support for establishing an efficient, stable, and sustainable management system for natural reserves.

## 2 Materials and methods

### 2.1 Sample preparation

This research employed artificial pruning to simulate varying grazing intensities in *C. tibetica*. Specifically, segments equivalent to 25%, 50%, and 75% of the aboveground plant parts were pruned, and plant samples measuring 2.0-2.5 centimeters from the top were collected as experimental samples representing grazing intensities of 25%, 50%, and 75%. The control group remained unpruned, and samples were directly obtained from the top 2-2.5 centimeters. Each grazing intensity consisted of six biological replicates. Plant material was gathered 48 hours post-pruning for subsequent physiological, biochemical, and metabolomics analyses. To ensure sample freshness during subsequent tests, the collected samples were promptly refrigerated in a mobile cooler and transported to the laboratory within 48 hours for analysis.

### 2.2 Physicochemical index detection

Soluble Protein Content (SPC) (Deans et al., 2018): Following the collection of plant samples, they were chopped and placed into 50 mL centrifuge tubes. Subsequently, 10 mL of Tris-HCl buffer was added, and the samples were dispersed using an ultrasonic instrument. After centrifugation to remove cellular debris, different concentrations of BSA standards and Bradford reagent were added to standard and test sample tubes, with a reaction time of 25 minutes. Absorbance was measured at 595 nm using a spectrophotometer, and the protein concentration in the test samples was calculated using a standard curve.

Catalase (CAT) activity (Mhamdi et al., 2010): Plant samples were diluted to a suspension, and a reaction mixture was prepared by combining diluted sample extracts, phosphate buffer, KCoCl_2_, and H_2_O_2_ in a test tube. The catalysis of H_2_O_2_ by catalase was initiated, and the reaction was stopped to observe bubble formation. Absorbance at wavelengths between 240-290 nm was measured using a spectrophotometer to assess catalase activity. Quantitative determination was conducted by comparing absorbance data with a standard curve.

Malondialdehyde (MDA) content (Davey et al., 2005): Plant leaf samples were heated in phosphoric acid buffer, and absorbance at 532 nm was measured using a spectrophotometer. MDA content was calculated using a standard curve or known MDA standards.

Peroxidase (POD) activity (Yoshida et al., 2003): The extraction of plant samples was combined with H_2_O_2_ and PBA solution, and absorbance at 470 nm was measured using a spectrophotometer to evaluate POD activity. Quantitative determination was achieved by comparing absorbance values with a standard curve or known POD activity of standard samples.

Proline (PRO) content (Lehmann et al., 2010): After centrifugation to remove sediment, proline was precipitated with TCA and re-dissolved. The reaction with a diethylamine reagent at 60°C was used to calculate proline concentration based on a standard curve.

Superoxide dismutase (SOD) activity: The reaction of sample extract with NBT solution, xanthine oxidase solution, NaH_2_PO_4_ solution, and Na_2_AsO_2_ solution was conducted under light conditions. After stopping the reaction, absorbance at 560 nm was measured using a spectrophotometer, and SOD activity was quantified by calculating from a standard curve.

### 2.3 Plant metabolomics detection

#### 2.3.1 Mass spectrometry

Triple TOF5600 MS & ExionLC AD LC (AB SCIEX); BEH C18 column (Waters); JXDC-20 nitrogen blow concentrator (Shanghai Jingsheng); LNG-T88 centrifugal dryer (Huamei Biochemical); SBL-10TD ultrasonic cleaner 300W-10L (Ningbo Xin Zhi); Centrifuge 5430R high-speed low-temperature centrifuge (Eppendorf); NewClassic MF MS105DU electronic balance (Mettler).

#### 2.3.2 Sample preparation

Metabolomic analysis was conducted on 64 sterilized yogurt samples from section 2.1. Approximately 50 mg of solid or 100 μL of liquid samples were placed in 1.5 mL centrifuge tubes and added with 400 μL of extraction solution (acetonitrile: methanol = 1:1). After vortex mixing for 30 s, low-temperature ultrasonic extraction was performed for 30 min (5°C, 40 KHz), followed by settling at -20°C for 30 min, centrifugation at 4°C, 13000 g for 15 min, removal of the supernatant, nitrogen drying, reconstitution with 120 µL reconstitution solution (acetonitrile: water = 1:1), low-temperature ultrasonic extraction for 5 min (5°C, 40 KHz), centrifugation at 4°C, 13000 g for 5 min, and transferring the supernatant to the injection bottle with an inner plug for subsequent analysis.

#### 2.3.3 Chromatography

Samples were separated using a BEH C18 column (100 mm × 2.1 mm i.d., 1.8 µm). Mobile phase A consisted of water (0.1% formic acid), while mobile phase B comprised acetonitrile/isopropanol (1:1) with 0.1% formic acid. The gradient elution was as follows: 0-3 min, linear change from 95% to 80% A and 5% to 20% B; 3-9 min, linear change from 80% to 5% A and 20% to 95% B; 9-13 min, 5% A and 95% B; 13.0-13.1 min, linear change from 5% to 95% A and 95% to 5% B; 13.1-16 min, 95% A and 5% B. Positive and negative ion scan mode within a mass range of m/z 50-1000. Ion spray voltage: positive 5000 V, negative 4000 V, declustering voltage 80 V, spray gas 50 psi, auxiliary heating gas 50 psi, curtain gas 30 psi, ion source heating temperature 500°C, collision energy ramping from 20 to 60 V.

#### 2.3.4 Data processing

LC-MS raw data were imported into the metabolomics software Progenesis QI (Waters) for baseline filtering, peak recognition, integration, retention time correction, peak alignment, generating a data matrix. The 80% rule was applied to remove missing values, fill gaps, total normalization, delete variables with QC sample RSD > 30%, log10 transformation, and matching with HMDB and Metlin databases.

### 2.4 Data analysis and visualization

#### 2.4.1 PCoA analysis

Utilizing the vegan package (version 2.6.2) in R software (version 4.2.0), the Bray-Curtis distance was computed, followed by unconstrained Principal Coordinates Analysis (PCoA) using the ade4 package (version 1.7.19) based on the Bray-Curtis distance.

#### 2.4.2 Differential metabolite analysis

The preprocessed data underwent differential metabolite analysis using the limma package (version 3.52.0). To identify differentially expressed genes (DEGs) between different sample groups, DESeq2 package was employed for differential expression analysis. Comparisons were made between the “CK” group and the “P25” group, “CK” group and “P50” group, as well as “CK” group and “P75” group. For enhanced data readability, column names were renamed as “CK_vs_P25”, “CK_vs_P50”, and “CK_vs_P75” to reflect comparisons between different groups. Metabolites were filtered based on the following criteria: P-value < 0.05 and FC > 2 defined an upregulated metabolite, while P-value < 0.05 and FC < -2 defined a downregulated metabolite.

#### 2.4.3 Metabolic pathway annotation

Metabolic pathway annotation was conducted using the KEGG database (https://www.kegg.jp/kegg/pathway.html) to identify pathways associated with differential metabolites. Pathway network visualization was executed using Release software (Version 2017-05-01). KEGG pathway enrichment analysis was performed via MetaboAnalyst online software (https://www.metaboanalyst.ca/). Python package scipy.stats was used for pathway enrichment analysis, acquiring the most relevant biological pathways through Fisher’s exact test associated with experimental treatments.

#### 2.4.4 Random forest analysis

In this study, to explore the relationship between metabolites and sample groups, a random forest classification model was employed. The randomForest package in R was used to establish this model, where the target variable was the experimental grouping, and the features were the expression data of metabolites. This model had the ability to identify important features and sample grouping. Initially, differential metabolite data between different sample groups were integrated into one data frame, aiding in narrowing the focus for better understanding of the data in subsequent analyses. Prior to analysis, data were normalized and scaled to ensure consistent scale and distribution. Various graphical tools like Variable Importance Plot, Scatterplots, and Heatmaps were employed to visually represent the model’s performance and outcomes. These graphics facilitated a better understanding of the relationship between metabolites and sample groups, providing an intuitive way to present important features and model performance.

#### 2.4.5 Metabolite correlation analysis

Based on the results from the random forest analysis, the top 30 metabolites according to MeanDecreaseGini were selected for correlation analysis. The R package Hmisc was used to compute Spearman’s correlation and corresponding P-values between these metabolites and physiological/biochemical indicators. Visualization of the computed results was done using the ggplot2 package (version 3.3.6).

## 3 Results

### 3.1 Physicochemical changes in *C. tibetica*

**Figure 1:**
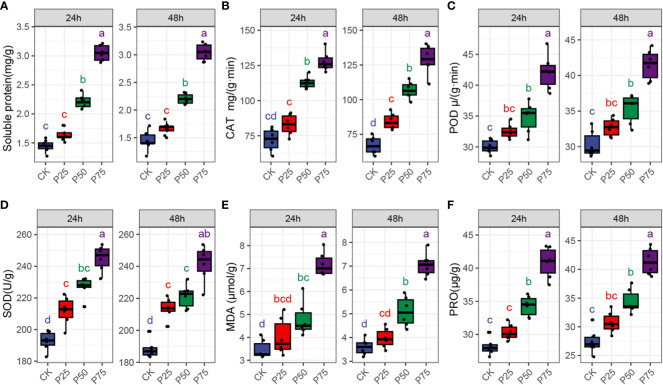
Comparison of plant protein and enzyme activity levels in Gallus gallus domesticus with different grazing intensities and at different times. **(A)** Soluble protein; **(B)**Catalase (CAT); **(C)** Malondialdehyde (MDA); **(D)** Peroxidase activity (POD); **(E)** Proline (PRO); **(F)** Superoxide dismutase activity (SOD).

To investigate the epigenetic differences in *C. tibetica* plants under different grazing intensities, this study assessed a series of physicochemical indicators, including soluble protein, peroxidase, malondialdehyde, peroxidation enzyme, proline, and superoxide dismutase (**Figure 1**). The analysis results indicated that, although no significant differences in soluble protein content were observed at different sampling times (24 and 48 hours) (*P>*0.05, **Supplementary Figure 1**), the soluble protein content gradually increased with the increase in grazing intensity within the same sampling time for branches. Particularly, in branches with grazing intensities of 50% and 75%, the protein content was significantly higher than that in the 25% grazing and control groups (*P<*0.05). This suggests that the increase in grazing intensity stimulates *C. tibetica* plants to secrete more enzyme agents, enhancing the metabolic activity of the plants. To further decipher the metabolic changes in plants, we examined the activities of peroxidase, peroxidation enzyme, and superoxide dismutase (**Figures 1B, D, F**; **Supplementary Figure 1**). The results showed that, within the same sampling time for branches, the enzymatic activities gradually increased with the increase in grazing intensity. Specifically, in branches with grazing intensities of 50% and 75%, the enzyme activities were significantly higher than those in the 25% grazing and control groups (*P<*0.05). This indicates that animal grazing activates the protective system of *C. tibetica*, enhancing the metabolic activity of the plants. It is noteworthy that we observed a significant increase in malondialdehyde content with the increase in grazing intensity. Especially in plants with 75% grazing intensity, the malondialdehyde content was significantly higher than in other groups (*P<*0.05). This suggests an increase in the extent of cell membrane damage in *C. tibetica* after grazing. Furthermore, within the same sampling time for branches, the free proline content gradually increased with the increase in grazing intensity. Particularly, in branches with 75% grazing intensity, the proline content was significantly higher than in the 25% grazing and control groups (*P<*0.05). These results demonstrate that with the increase in animal grazing intensity, the metabolic intensity of *C. tibetica* plants increases, various protective enzyme activities rise, indicating a synchronous increase in proline and malondialdehyde content, representing the plant’s resistance. In summary, animal grazing on *C. tibetica* increases its metabolic intensity, membrane lipid peroxidation, and resistance.

### 3.2 Non-volatile metabolite characteristics in *Caragana tibetica* under different grazing intensities

**Figure 2:**
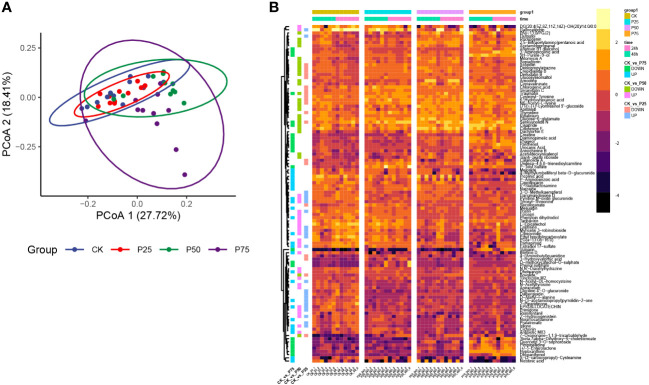
Metabolite characteristics of Gallus gallus domesticus plants at different grazing intensities. **(A)** Unconstrained PCoA plot based on Bray–Curtis distance. **(B)** Heatmap of the top 100 metabolites in terms of abundance.

In order to gain a more profound understanding of the metabolic dynamics and adaptive responses to varying grazing intensities in *C. tibetica*, this study conducted an analysis of non-volatile metabolites. Within 32 samples classified according to the KEGG Second Category, a total of 2303 compounds were identified (**Supplementary Table 1**). Results revealed a dynamic shift in the composition of non-volatile metabolites in response to changes in grazing intensity. Principal Co-ordinates Analysis (PCoA) utilizing Bray-Curtis distance exhibited distinct clustering along axes, indicating pronounced separation among samples from different grazing intensity groups (**Figure 2A**). These findings suggest that animal grazing intensity significantly influences the structural composition of plant metabolites, thereby reshaping the metabolic landscape of *C. tibetica*. Visualization of the top 100 metabolites by abundance (**Figure 2B**) highlighted substantial variations in metabolite content among samples with different grazing intensities. The heatmap clearly depicted differential metabolite profiles between groups: the control group exhibited significant differences with samples at 25% grazing intensity for 43 metabolites, at 50% grazing intensity for 47 metabolites, and at 75% grazing intensity for 55 metabolites. Notably, a noteworthy increase in Traumatin content was observed in *C. tibetica* plants post-animal grazing (*P<*0.05). Concurrently, as grazing intensity increased, the content of Epigallocatechin gradually declined. Overall, animal grazing exerted a significant impact on the metabolite composition of *C. tibetica* plants, leading to conspicuous alterations in key metabolites. This underscores the activation of defense mechanisms and heightened antioxidant capacity in *C. tibetica* following grazing.

### 3.3 Impact of grazing intensity on metabolism in *Caragana tibetica*

**Figure 3:**
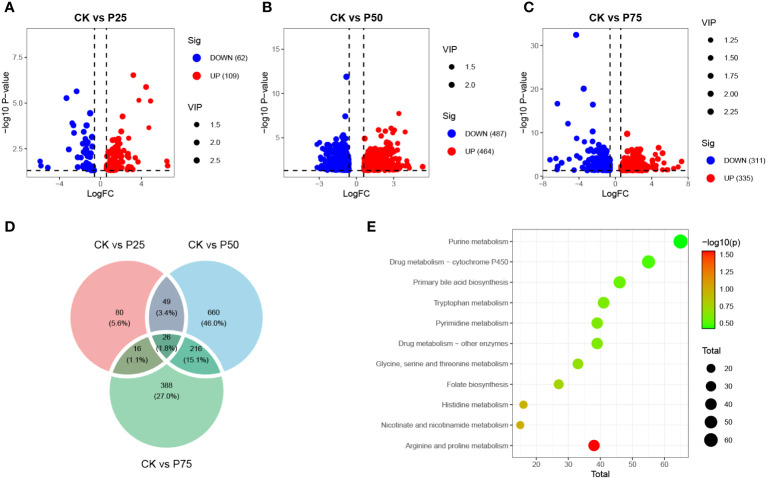
Differential metabolite landscape between different grazing intensities. The volcano plots show the differential metabolites between the CK group and the P25 group **(A)**, between the CK group and the P50 group **(B)**, and between the CK group and the P75 group **(C)**; **(D)** Venn diagram illustrates the overlap of metabolites among the three groups’ differential analysis results; **(E)** KEGG-enriched pathways for differential metabolites.

This study conducted differential metabolite analysis of simulated animal grazing intensities using metabolomics data. A volcano plot was generated to reveal significant differences in the metabolite composition under various grazing intensities. The results showed that, compared to the control group, samples subjected to 25% grazing intensity exhibited annotation for 171 different metabolites, including 109 upregulated and 62 downregulated metabolites (*P<*0.05, **Figure 3A**). Under the same significance threshold, samples subjected to 50% grazing intensity were annotated with a total of 951 different metabolites compared to the control group, with 464 upregulated and 487 downregulated metabolites (*P<*0.05, **Figure 3B**). In contrast, samples subjected to 75% grazing intensity were annotated with 646 different metabolites compared to the control group, with 335 upregulated and 311 downregulated metabolites (*P<*0.05, **Figure 3C**). These differential metabolites exhibited a correlation with the intensity of animal grazing behavior, revealing the metabolic responses of organisms to different grazing intensities. These findings contribute to understanding the physiological adaptability and regulatory mechanisms of metabolic pathways in *C. tibetica* under various grazing conditions. To further explore the common and unique associations among differential metabolites in treatment groups with different grazing intensities, a Venn diagram was constructed to visualize the overlap and uniqueness of metabolites between 0% vs. 25%, 0% vs. 50%, and 0% vs. 75% treatment groups (**Figure 3D**). The results showed that 1.8% of metabolites were commonly shared among the three groups of differential metabolites, suggesting that these metabolites may have similar importance or biological significance for organisms exposed to multiple grazing intensities. Through annotation with the KEGG database, we conducted enrichment analysis of biological functions and metabolic pathways for both shared and unique differential metabolites (**Figure 3E**). These metabolites were found to be involved in Purine metabolism, Tryptophan metabolism, and Pyrimidine metabolism pathways.

### 3.4 Machine learning algorithm identifies core metabolites

**Figure 4:**
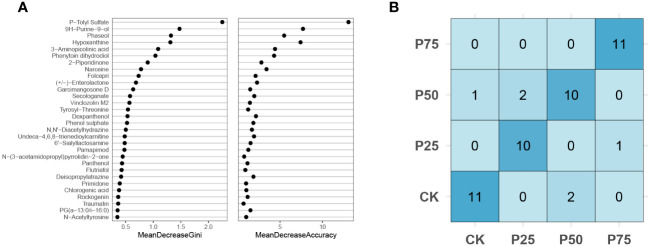
Random forest analysis of signature metabolites in Gallus gallus domesticus plants at different grazing intensities. **(A)** Confusion matrix; **(B)** MeanDecreaseGini index and MeanDecreaseAccuracy for the top 30 core metabolites.

This study employed the random forest classification (RFC) algorithm to unveil the core metabolite species significantly influencing the stress resistance process in *C. tibetica*. Through the analysis of extensive datasets, this algorithm identifies the most crucial metabolites in stress resistance, providing a deeper understanding of how these metabolites impact the plant’s resilience (Tripathi et al., 2021). The RFC model was applied to analyze metabolomic data under simulated animal grazing intensities (0%, 25%, 50%, 75%). The model identified the top 30 differentially significant metabolites as key features, aiming to explore the impact of animal grazing behavior on the composition of metabolites (**Figure 4A**). MeanDecreaseAccuracy and MeanDecreaseGini were two feature importance evaluation metrics utilized to assess the contribution of features to model performance and split nodes. According to the classification results, P-Tolyl Sulfate exhibited the highest MeanDecreaseAccuracy and MeanDecreaseGini. P-Tolyl Sulfate is commonly found in soil, water, and air, serving as a pollutant. This suggests that as grazing intensity increases, the level of soil contamination affecting *C. tibetica* plants also rises. The top 10 differentially significant metabolites, such as Narceine, Phaseol, and Hypoxanthine, provide crucial clues to understanding the impact of grazing behavior on the metabolic composition of *C. tibetica*, laying the foundation for further research on the correlation between metabolic reactions within organisms and behavior. The confusion matrix is a crucial tool for evaluating the performance of classification models. In this study, the RFC model predicted simulated animal grazing intensity, and a confusion matrix was constructed based on the prediction results to assess the model’s performance and reliability (**Figure 4B**). The analysis of the confusion matrix displayed the predictive performance of the model across different grazing intensity categories. Performance metrics derived from the confusion matrix evaluated the overall accuracy and predictive capabilities for different categories. The results indicated accuracy exceeding 83.3% across all groups, demonstrating the high predictive efficacy of core metabolites for *C. tibetica* under varying grazing intensities. These findings further support the association between core metabolites and the response of *C. tibetica* to grazing intensity changes, highlighting the high predictive accuracy of the RFC model constructed using these metabolites.

### 3.5 Correlation between core non-volatile metabolites and biochemical indices in *Caragana tibetica*

**Figure 5:**
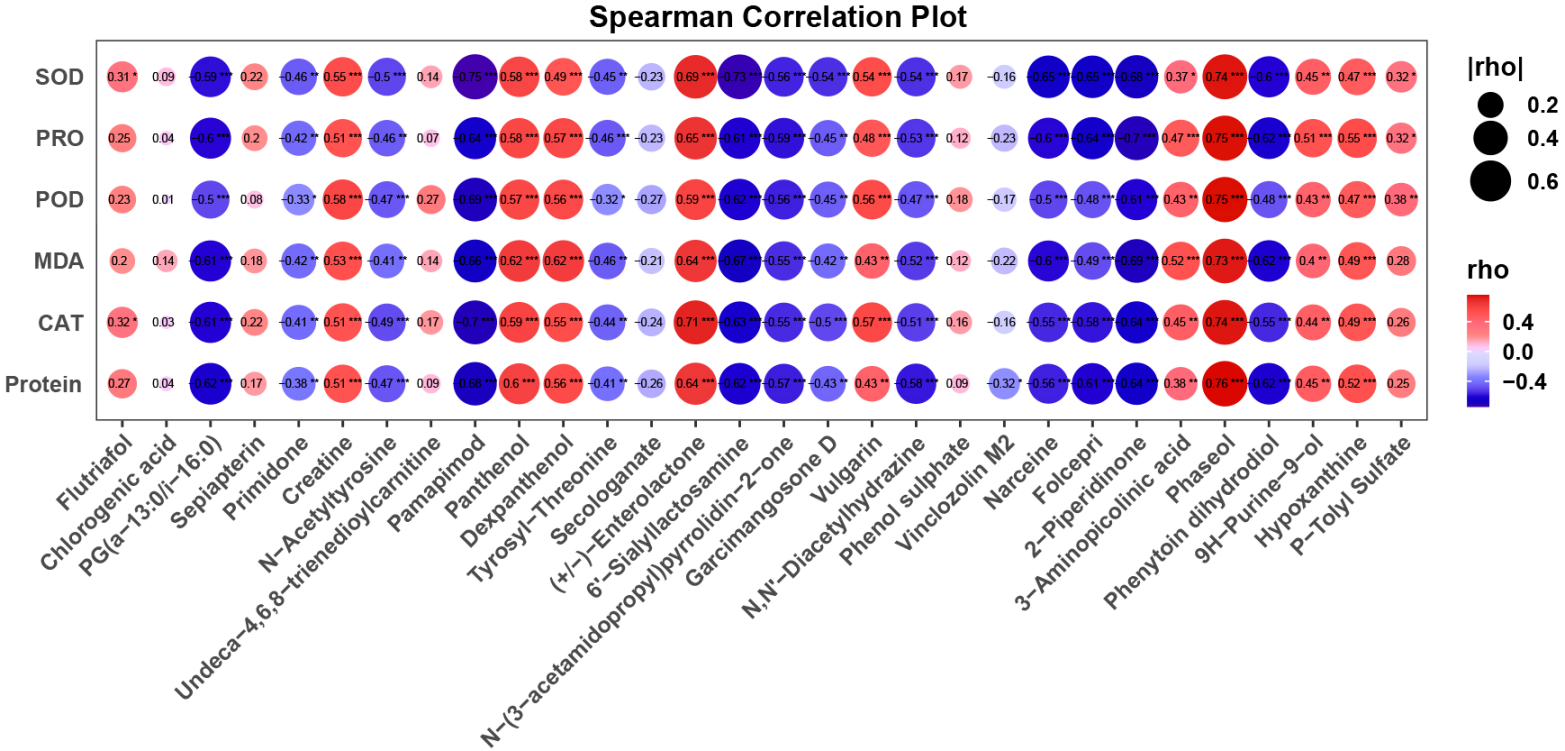
Bubble plot showing the correlation between core compounds and epigenetic indicators, with bubble size and color representing the correlation coefficient. The font and “*” within the bubbles indicate the correlation coefficient and p-value.

To elucidate the intricate correlation between core metabolites and biochemical indicators in *C. tibetica*, this study conducted a correlation analysis between the previously identified 30 metabolites and data on proteins, enzyme activities, and other parameters (**Figure 5**). Initially focusing on the connection between metabolomic data and enzyme activities as well as protein content, it was observed that five metabolites exhibited a significant positive correlation (*P <* 0.05, r > 0.6) with CAT activity, SOD activity, POD activity, and soluble protein content. Simultaneously, fifteen metabolites showed a significant negative correlation (*P >* 0.05, r > 0.6). These findings suggest that these specific metabolites may play a role in crucial biochemical pathways closely associated with enzyme activity. Further analysis unveiled that two metabolites displayed a significant positive correlation (*P <* 0.05, r > 0.6) with PRO, while six metabolites exhibited a significant negative correlation (*P >* 0.05, r > 0.6). Metabolites such as Pamapimod and Sialyllactosamine, negatively correlated with proline levels, hint at a potential association of their accumulation with proline metabolic pathways. Lastly, the study explored the link between metabolomic data and MDA levels. MDA, a critical oxidative stress indicator, is related to plant stress responses and redox balance. Four metabolites were identified with a significant positive correlation (*P <* 0.05, r > 0.6) with MDA, while five metabolites showed a significant negative correlation (*P >* 0.05, r > 0.6).

### 3.6 Variation in content of core non-volatile metabolites with grazing intensity

**Figure 6:**
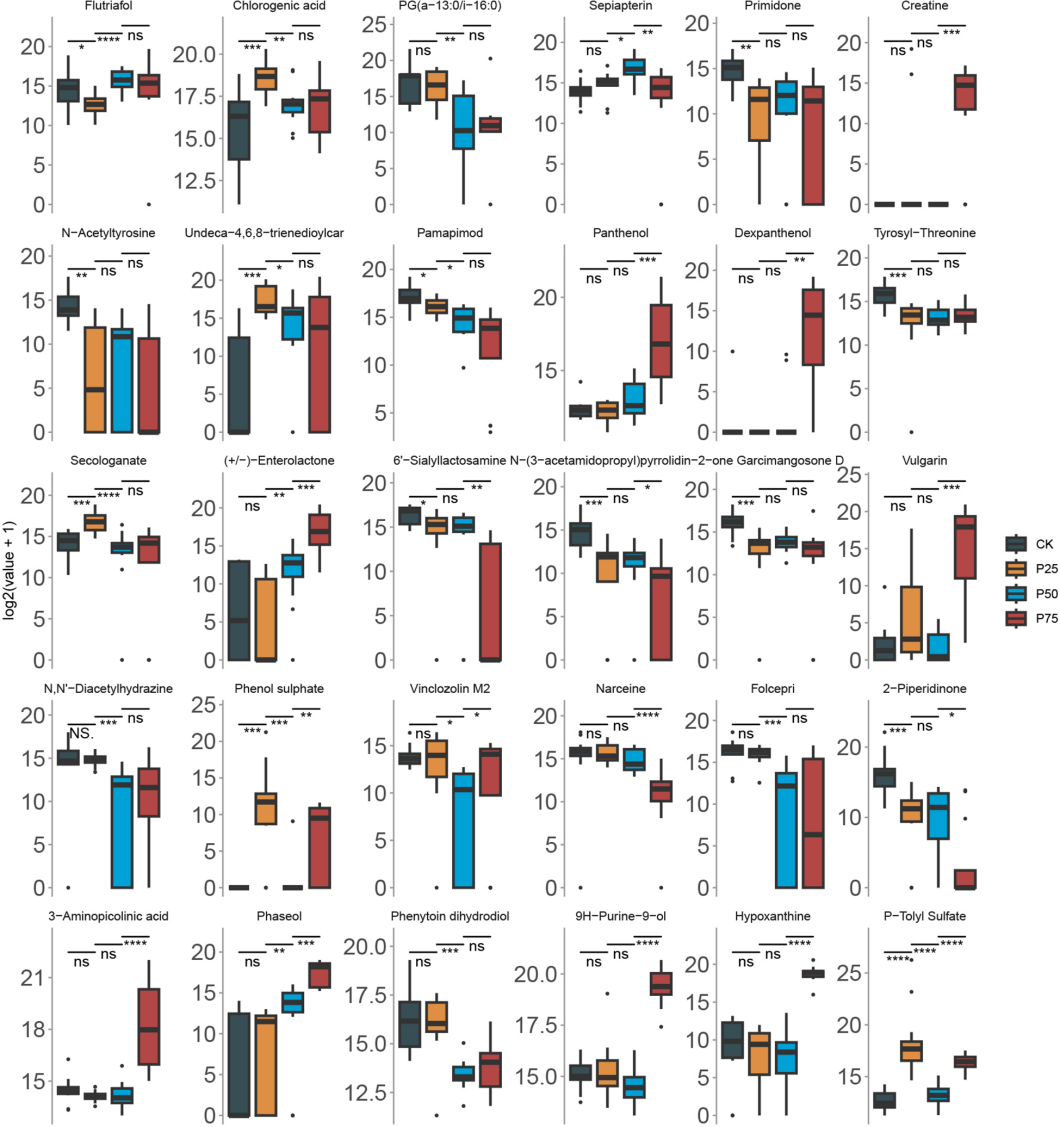
Comparison of core metabolite levels in Gallus gallus domesticus at different grazing intensities. The wilcox-test significance markers: ns, p > 0.05, ""p ≤ 0.05, ""p ≤ 0.01, ""p ≤ 0.001, "****"p ≤ 0.0001.

In the preceding section, we utilized metabolomic data and the analysis from the RFC model to select the 30 most important metabolites based on their importance scores in the model. Subsequently, we delve into the variations in the content of these substances among different sample groups (**Figure 6**). Our results indicate significant differences in the content of these 30 core metabolites, and these differences have statistical significance across various sample groups. The variations in these substances may reflect the metabolic responses of *C. tibetica* under different physiological states. We observed a significant increase in the levels of Enterolactone and Panthenol among different groups (*P<*0.05). This gradual increase may reflect the adaptability of *C. tibetica* to grazing, responding to changes in the environment or alterations in the metabolic demands within the organism. Furthermore, substances such as Creatine, Dexpanthenol, Vulgarin, and 3−Aminopicolinic acid were found to have significantly higher levels in the sample group with a grazing intensity of 75% compared to other groups (*P<*0.05). These findings imply the complexity of plant metabolism, involving not only the synthesis of substances but also their breakdown and regulation. Higher grazing intensity may impose environmental pressure on plants, leading to significant changes in metabolite levels. The increase in these substances may represent a physiological response of *C. tibetica* to environmental stress.

## 4 Discussion

Malondialdehyde is a byproduct of lipid peroxidation in plants under stress conditions, and its concentration reflects the intensity of membrane peroxidation, with higher values indicating more severe damage to the plant’s cell membrane system (Mas-Bargues et al., 2021). The level of free proline is a crucial indicator for assessing plant stress resistance, and numerous studies have shown that free proline is involved in the stress-induced biochemical activities of most plants (Lehmann et al., 2010). According to the findings of this study, the concentrations of malondialdehyde and proline significantly increased with an increase in grazing intensity, indicating an increase in the damage to the cell membrane of *C. tibetica* after grazing. Soluble proteins play a key role in plant growth and are vital components of many enzymes that reflect the overall metabolism of the plant (Kučerová et al., 2019). We also found that there was no significant difference in the soluble protein content among branches sampled at different times. However, with an increase in grazing intensity, branches sampled at the same time exhibited an increase in soluble protein content.

Catalase is one of the important protective enzymes in plants, responsible for clearing hydrogen peroxide within the plant to protect cells from damage by reactive oxygen species (Karuppanapandian et al., 2011). Peroxidase is also a widely distributed antioxidant enzyme in plants, serving to eliminate H_2_O_2_ generated during plant metabolism and alleviate oxidative damage caused by reactive oxygen species (Ma et al., 2008). Superoxide dismutase is a critical protective enzyme in plants, catalyzing the conversion of superoxide anion O^2-^ to H_2_O_2_ to eliminate the harm caused by O^2-^ to cells (Ighodaro and Akinloye, 2018). Animal grazing increased the activities of catalase, peroxidase, and superoxide dismutase in *C. tibetica* plants. These results suggest that grazing activates the plant’s protective system, enhancing its metabolic processes. Research by Ma et al. found higher SOD and POD activities in *C. tibetica* in the desert, indicating that this species possesses stronger antioxidant capabilities and can more effectively cope with oxidative stress in harsh environments (Ma et al., 2008). Overall, animal grazing increases the metabolic intensity, membrane peroxidation, and stress resistance of *C. tibetica*.

Traumatin is a metabolite associated with plant stress resistance and defense mechanisms. Its synthesis or accumulation in plants is often considered a response to various environmental stressors, such as insect attacks, pathogen infections, drought, and UV radiation (Bonner and English, 1938). Anna et al.’s research revealed that traumatic acid, a non-enzymatic oxidation product of traumatin, plays a crucial role in regulating the growth and metabolism of *Wolffia arrhiza*. Traumatic acid may be involved in the activation of antioxidant enzymes and the metabolic response of low-water plants to oxidative stress (Pietryczuk and Czerpak, 2012). Similarly, in *C. tibetica* plants, we observed a significant increase in traumatin content after grazing, indicating the activation of defense mechanisms. Epigallocatechin is a flavonoid metabolite commonly found in camellia shrubs. Some studies suggest that epigallocatechin may influence plant growth and development (Tanaka et al., 2002). This could be related to the physiological adaptation and regulation of plants in response to environmental stress. Changes in epigallocatechin content in samples with different grazing intensities may be associated with the adaptive growth of *C. tibetica*.

This study primarily analyzed the changes in metabolic pathways in *C. tibetica* after grazing. Our results show a significant correlation between differential metabolites and the purine metabolism pathway. Literature indicates that purine metabolism is involved in providing energy to plants, and synthesized nucleotides (ATP and GTP) serve as essential energy molecules in plant cells. When facing environmental stress, plants require more energy to execute defense and adaptation strategies (Stasolla et al., 2003). We observed that the synthesis of differential metabolites is linked to tryptophan metabolism. Tryptophan is a precursor to hormones (such as auxins, melatonin, and gibberellins) crucial for various growth processes in plants (Arnao and Hernández-Ruiz, 2015). By regulating plant growth and development, these hormones help plants cope with environmental stress (Radwanski and Last, 1995). Similarly, we found a correlation between differential metabolites and pyrimidine metabolism. Pyrimidine is a fundamental component of DNA and RNA. Plants may need to synthesize more DNA and RNA when subjected to various environmental stresses for repair, growth, or stress response (Tuteja et al., 2001).

We identified P-Tolyl Sulfate as the most crucial feature in the RFC model. P-Tolyl Sulfate is typically considered a pollutant and may be present in soil, water, and air. Although plants may be influenced by environmental pollutants, such metabolites usually exert harmful effects on plants (Nicolis et al., 2009), unrelated to their stress resistance. Simultaneously, core metabolites from the RFC model include narceine. Narceine is an alkaloid, and alkaloids, as a class of metabolites, often possess toxicity, serving as defenses against natural enemies of plants, such as insects and herbivores (Chowdhury et al., 2005). Narceine may play a role in the self-defense mechanism of *C. tibetica*, reducing plant consumption or damage. Research by Chowdhury et al. indicates that narceine, a potent alkaloid isolated from Corydalis longipes, effectively inhibits spore germination of certain plant-pathogenic fungi at low concentrations (Chowdhury et al., 2005). These results suggest that core metabolites validate the impact of grazing intensity changes on *C. tibetica*, while the RFC model built with these metabolites accurately predicts grazing intensity.

According to Stitt et al.’s research, specific metabolites may participate in regulating crucial biochemical processes such as redox balance, energy metabolism, and substance transport in plants, thereby influencing enzyme activity (Stitt et al., 2010). Our research reveals a significant association between core non-volatile metabolites and *C. tibetica*’s enzyme activity, providing a novel perspective for a deeper exploration of plant physiology and metabolism. Proline plays a vital role in plant growth and biochemical processes, and this study found a significant correlation between Pamapimod, Sialyllactosamine, and proline levels, suggesting that these metabolites may be involved in regulating the proline metabolic pathway, which has crucial implications for understanding the growth and nitrogen metabolism of *C. tibetica*. Furthermore, the correlation between metabolites and MDA levels indicates a crucial role for these metabolites in stress response, potentially regulating MDA levels to help *C. tibetica* cope with environmental stress. Researchers have found both free and bound MDA in plant samples, observing a significant increase in free MDA under oxidative stress conditions (Weber et al., 2004). Moderate grazing intensity can aid in the recovery and regeneration of *C. tibetica*.

Enterolactone is a potent antioxidant that helps neutralize oxidative stress within cells. When exposed to environmental stressors such as high temperatures, drought, or ultraviolet radiation, plants may actively produce antioxidant substances to protect cells from oxidative damage (Heinonen et al., 2001). Panthenol is a compound belonging to the vitamin B family and participates in the synthesis and reinforcement of plant cell walls. The cell wall serves as the primary defense line for plants, resisting pathogen invasion. Strengthening the cell wall contributes to enhancing the plant’s resistance to pathogens and harmful microorganisms (Zhu et al., 2023). We observed an increase in Enterolactone and Panthenol levels with increasing grazing intensity, suggesting that these substances may participate in the plant’s adaptive response to resist external pressure or cope with environmental changes. On the other hand, some substances such as Pamapimod, Undeca−4,6,8−trienedioylcar, Narceine, and Folcepri exhibited significantly decreased levels among different sample groups. These substances may no longer be needed in specific physiological states, or their metabolic pathways may be negatively regulated. Narceine, mentioned earlier, is an alkaloid that defends plants against avian and herbivorous predators. The decrease in narceine content indicates a weakening of this defensive capability with increasing grazing intensity. Overall, our study reveals significant differences in core metabolites among different groups, reflecting the metabolic responses of *C. tibetica* in different physiological states. These results provide valuable clues for further research to delve into the roles of these core substances in the physiology and stress resistance of *C. tibetica*.

## 5 Conclusion

Grazing exacerbates cellular damage in *C. tibetica*, significantly alters the plant’s metabolic profile, and activates its antioxidative and stress-responsive biochemical mechanisms. Specific metabolites participate in *C. tibetica’s* stress response through multiple metabolic pathways, correlating with the antioxidative enzymes and damage repair of *C. tibetica* branches. *C. tibetica* enhances the synthesis of these substances to adapt to various challenges. Moderate grazing intensity facilitates Tibetan Snowcocks in bolstering the synthesis of essential metabolites, thereby enabling adaptation to diverse challenges and fostering regeneration. This study provides further insights and potential solutions for the sustainable growth of *C. tibetica*.

## Data availability statement

The original contributions presented in the study are included in the article/**Supplementary Material**. Further inquiries can be directed to the corresponding author.

## Author contributions

MH: Data curation, Investigation, Methodology, Visualization, Writing – original draft, Writing – review & editing. YH: Investigation, Validation, Visualization, Writing – original draft, Writing – review & editing. YG: Project administration, Writing – original draft. MH: Investigation, Methodology, Visualization, Writing – review & editing. LD: Funding acquisition, Project administration, Resources, Supervision, Writing – review & editing.
