# Supplementary figures and images for: Decoding the metabolomic responses of Caragana tibetica to livestock grazing in fragile ecosystems
Source: Front Plant Sci. 2024 Feb 26;15:1339424. doi: 10.3389/fpls.2024.1339424 (PMC10959174; doi:10.3389/fpls.2024.1339424)

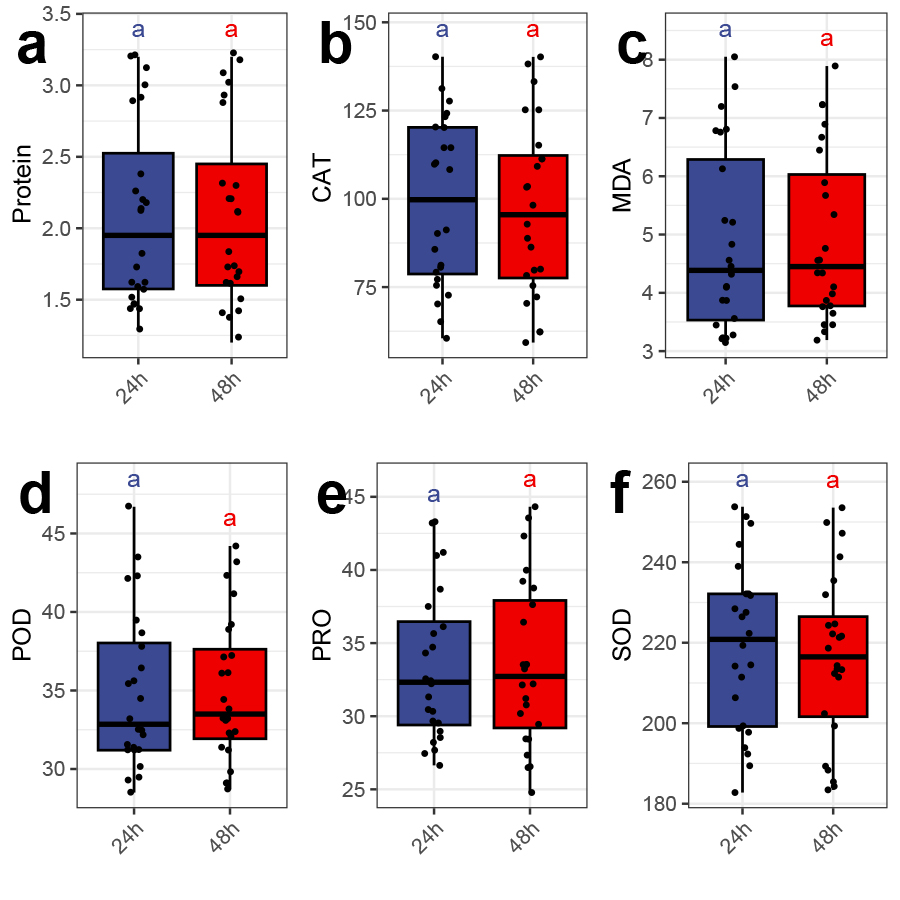

Supplement: Supplementary file 1 [file Image_1.jpeg]
